# Tuberculosis Topics

**Published:** 1918-12-14

**Authors:** 


### TUBERCULOSIS TOPICS.

### Remedies for Sanatoria Shortage.

Nothing much fresh falls as yet to be chronicled. The old cry of the long waiting-list for sanatorium treatment of civilians continues to be heard, as at the last monthly meeting of the Staffordshire Insurance Committee, and in the West Riding of Yorkshire, where it is said there are 160 cases waiting. However, some hope is derived from the action of the Local Government Board in promising Treasury grants of four-fifths of the capital outlay on extensions of existing institutions. The President of the Board has recently conferred with the Minister of Pensions and representatives of the National Health Insurance Commission on the shortage of residential accommodation for the treatment of tuberculosis. The outcome is a series of rather platitudinous recommendations. Cannot the accommodation for infectious diseases be further drawn upon, subject, of course, to the condition that the tuberculous patients would be promptly removed in the event of the accommodation being required for its original purpose? The answer to this is that such accommodation was already being drawn upon four years ago. "Poor-Law accommodation structurally separate from other such accommodation," is also indicated as a resource; but naturally this is very scanty. It is gravely pointed out also that existing buildings are available for the housing of tuberculous patients more quickly than

**Tuberculosis Topics**—(*continued*).

those not yet erected; as also that Councils should satisfy themselves that the water supply and the drainage arrangements are adequate for the number to be accommodated. New buildings are deprecated on the score of expense. Authorities will reflect that as yet they are practically impossible for want of material and labour.

**Wages for Consumptive Colony Patients.**

Patients' complaints are again a feature of local newspapers. The Local Government Board was weak enough to endorse one to the effect of absence of medical examination for twenty-five days; the man apparently had been examined on the 4th and 29th of April. It is of course obvious to all of real sanatorium experience that once a month is ample for routine examination. The County and Borough Councils and Insurance Committees in Northumberland and Durham are contemplating the purchase of an estate large enough for the needs of the two counties, and the provision of 100 beds, at a capital cost of £25,000 (or alternatively £15,000), or to provide sixty beds at £12,500. This is for a farm and industrial colony for consumptives in the area. The payment of wages is a feature of the scheme. The present funds were insufficient, but assistance was expected from the Insurance Commissioners. After discussion, a resolution was carried recommending to the local authorities the scheme suggested.

